# Optogenetic sleep enhancement improves fear-associated memory processing following trauma exposure in rats

**DOI:** 10.1038/s41598-020-75237-9

**Published:** 2020-10-22

**Authors:** Christopher J. Davis, William M. Vanderheyden

**Affiliations:** Department of Biomedical Sciences, Pharmaceutical and Biomedical Sciences, WSU Health Sciences Spokane, Elson S. Floyd College of Medicine, Room 213/Lab 230, 412 E. Spokane Falls Blvd, Spokane, WA 99202 USA

**Keywords:** Optogenetics, Preclinical research, Stress and resilience

## Abstract

Sleep disturbances are commonly found in trauma-exposed populations. Additionally, trauma exposure results in fear-associated memory impairments. Given the interactions of sleep with learning and memory, we hypothesized that increasing sleep duration following trauma exposure would restore overall function and improve trauma-induced fear-associated memory dysfunction. Here, we utilized single prolonged stress, a validated rodent model of post-traumatic stress disorder, in combination with optogenetic activation of hypothalamic melanin-concentrating hormone containing cells to increase sleep duration. The goal of this work was to ascertain if post-trauma sleep increases are sufficient to improve fear-associated memory function. In our laboratory, optogenetic stimulation after trauma exposure was sufficient to increase REM sleep duration during both the Light and Dark Phase, whereas NREM sleep duration was only increased during the Dark Phase of the circadian day. Interestingly though, animals that received optogenetic stimulation showed significantly improved fear-associated memory processing compared to non-stimulated controls. These results suggest that sleep therapeutics immediately following trauma exposure may be beneficial and that post-trauma sleep needs to be further examined in the context of the development of post-traumatic stress disorder.

## Introduction

Post-traumatic stress disorder (PTSD) arises as a result of experiencing a physical or psychological trauma and is often accompanied by significant sleep disorders^1^. In fact, as many as 70–91% of PTSD patients have difficulty falling asleep and staying asleep through the night^2,3^. Furthermore, poor sleep has been linked to the exaggeration of PTSD symptoms such as anxiety, flashbacks, agitation, and fear^4–11^. Humans resilient to PTSD show increased theta activity during rapid eye movement (REM) sleep^10^ and reduced autonomic nervous system activation as measured by changes in heart rate during quiescent periods^12,13^. These data suggest a reciprocal relationship between sleep and PTSD wherein trauma exposure impairs sleep and sleep disorders increase the severity of PTSD^14^. We hypothesize that sleep interventions designed to increase post-trauma sleep will break this vicious cycle and help stave off the development of PTSD.

Examining trauma-exposure-dependent sleep disturbances in humans is prohibitively difficult because the timing and intensity of trauma exposure is unpredictable. Therefore, to examine the molecular and systems biology components of trauma-exposure-dependent sleep disturbances, a pre-clinical animal model is required^15^. *Single prolonged stress* (SPS) is a rodent model of PTSD that consists of a combination of stressors that include physical restraint, a 20 min forced swim, exposure to ether vapors (a potent chemical activator of the stress system) until anesthetized, and seven days of social isolation^16^. SPS causes molecular, anatomical, and behavioral changes that resemble PTSD in humans^16–18^. Rats that have been exposed to SPS show fear-associated memory impairments including enhanced fear-renewal and deficits in extinction-retention for both contextual and cued fear^19,20^. These fear-associated memory impairments are used as a metric to determine the severity of the PTSD phenotype in rodents. Importantly, SPS results in quantifiable and robust sleep disturbances in rats^8,17^, and has repeatedly shown good face validity to the human condition of PTSD^18^. Thus, SPS is an ideal animal model to assess the mechanisms regulating trauma-exposure-dependent sleep disorders. Further, fear-associated memory tests are a functional metric for confirmation of PTSD in this pre-clinical PTSD model.

In our previous work, we examined the effect of SPS exposure at Zeitgeber time (ZT) 0, when nocturnal rodents are predominantly asleep^17^. However, a gap in the literature exists to explain how the circadian system and the timing of trauma affects subsequent trauma-dependent sleep and fear-associated memory alterations. How SPS exposure alters sleep when animals receive SPS at the beginning of their predominantly awake period, ZT12, was assessed in our first set of experiments.

The precise role of sleep in mediating, exacerbating, or mitigating negative health outcomes in trauma exposed populations is unknown. Therefore, the main goal of this work was to assess the effects of post-trauma sleep increases on the development of PTSD phenotype in SPS exposed rats. Sleep manipulations, specifically increasing sleep duration, in rodents has become increasingly easier due to the development of optogenetic tools^21,22^. Optogenetic stimulation of melanin-concentrating hormone (MCH) cells within the lateral hypothalamus (LH) results in increased REM and non-rapid eye movement (NREM) sleep in mice^23,24^ and rats^25^. Therefore, in our second set of experiments, we used post-SPS stimulation of MCH cells to increase sleep duration and then assessed fear-associated memory function to determine the contribution of sleep increases to the severity of the PTSD-like phenotype in control and optogenetically stimulated animals.

## Methods

### Animals

All animal procedures were carried out in accordance with the National Institutes of Health *Guide for the Care and Use of Laboratory Animals* and approved by the *Washington State University Committee on the Use and Care of Laboratory Animals.*

Male, Long Evans rats (60–90 days old) were housed in temperature and humidity-controlled rooms and given ad libitum access to food and water. The animals underwent two surgeries, the first surgery was performed at 60–65 days old to stereotaxically inject the experimental or the control viral vector. Two weeks following the viral injection, animals underwent a second surgery to implant optogenetic stimulating fiber optic cable (optrodes) and electroencephalographic (EEG)/electromyographic (EMG) recording electrodes (described below). Animals were housed singly following surgical procedures.

### Viral vector

We generated a plasmid containing the Channelrhodopsin-2 gene (ChR2) with an enhanced yellow fluorescent protein (EYFP) marker under the melanin-concentrating hormone (MCH) promoter sequence as previously published in Blanco-Centurion^5^. This MCH specific plasmid was packaged into a recombinant adeno-associated viral vector (rAAV, serotype 5) by the Vector Core at the University of North Carolina (Chapel Hill, NC) to create rAAV-MCH-ChR2-EYFP. Additionally, a control vector lacking the ChR2 gene (rAAV-MCH-EYFP) was constructed as previously published^26^.

### Viral transfection

Rats underwent surgery to inject viral vectors into the LH. Surgeries were performed with the aid of a digital stereotaxic apparatus (Stoelting, Wood Dale, IL), using aseptic conditions, under constantly monitored isoflurane anesthesia (1.5–2%). A midsagittal incision was made to reveal the skull which was cleaned and prepped using hydrogen peroxide solution and saline. Holes were drilled in the skull above the LH and rAAV viral vectors were microinjected bilaterally targeting the MCH neurons of the LH (AP =  − 2.9 mm, lateral =  ± 1.2 mm, vertical =  − 7.8 mm). Virus (3 µl per hemisphere over 20 min) was delivered using microinjection pumps (Stoelting, Wood Dale, IL) and 10 µl Hamilton (Reno, NV) syringes. The injection needle was left in place for an additional 5 min before being removed. The incision was sutured closed at the conclusion of the surgery. Animals were treated with Dexamethasone (200 µg/kg), ciprofloxacin (5 mg/kg) and buprenorphine SR (1 mg/kg) to manage swelling, infection, and pain, respectively. Post-mortem studies were conducted to validate expression of EGFP as previously published^25^. Histological verification of expression of the viral construct was made by examining 4% paraformaldehyde perfused, frozen, and cryostat sectioned (40 micron) tissue using a Zeiss ApoTome fluorescent microscope. Histological analysis revealed similar anatomical expression to previously published data^25^ and a representative image is shown in Figure S1.

### Electrophysiology/optogenetics

Two weeks after the rAAV surgery, a second stereotaxic surgery was performed to implant the EEG/EMG sleep recording electrodes and stimulating optrode into the LH. As above, aseptic surgeries were performed under isoflurane anesthesia. A midsagittal incision was made on the top of the skull and the skin was retracted. After cleaning the surface of the skull, 2 holes were drilled through the cranium and screw electrodes (Plastics One, Roanoke, VA) were inserted bilaterally over the frontal area (± 2.5 mm lateral to midline, 2.5 mm anterior to Bregma) for EEG recordings. Two flexible wire electrodes were threaded through the dorsal neck muscles for EMG recordings. Gold pins were connected to the ends of each electrode then placed into a six-pin connector (Plastics One, Roanoke, VA) which was attached to the skull via light-curable dental acrylic. The holes in the skull that were previously used for injection of the rAAV were re-drilled to accommodate the insertion of a custom 3D printed head piece that housed guide cannula for placement of the optrode. The fiber optic unit was lowered through the guide cannula before finalizing the electronic connections through the six-pin connector to the Tucker Davis Technology (TDT) (Alachua, FL) electrophysiology recording device and TTL controller. Rats were given at least 10 days to recover from surgery prior to beginning the experiment. Animal well-being was assessed daily during the surgical recovery period. Any sign of illness or pain, including decreased motility and responsiveness, vocalizations, lack of appetite, decreased grooming, etc. was noted and treated in consultation with veterinary staff.

#### Optogenetic stimulation

Blue light was delivered by bilateral head mounted blue-light-emitting LEDs (Broadcom, HSMR-C191-S0000) driven at 5v, at a frequency of 10 Hz for one minute during every 5 min period. The rate of stimulation was controlled by a TTL program developed in-house for the TDT system. Rats were stimulated for 7 consecutive days during the incubation period of SPS.

#### Sleep recording and analysis

As previously reported^17^, following recovery from surgery, animals were housed individually and connected to the TDT recording system via a lightweight, flexible tether attached to a commutator (Sparkfun.com, Slip Ring) for free movement within the cage. The recording system was used to sample signals at 333 Hz, filtered between 0.1 and 100 Hz and amplified. Prior to analysis, signals were down-sampled to 250 Hz. The two EEG electrodes were differentially referenced to obtain one EEG channel. Two EMG channels were also differentially referenced to obtain the EMG signal. Animals were given 48 h to acclimate to the tethers prior to beginning baseline recordings. During the acclimation period, animals were supplied food to last the duration of the EEG/EMG experimental recordings. While connected to tethers, animals were monitored daily for food, water, and health via visual inspection and through the use of a video monitoring system to avoid disturbing the animals.

Collected data were transferred from the recording PC, stored onto disk, and scored off-line in 10-s epochs to determine sleep/waking state using Sleep Sign software (Nagano, Japan). Three vigilance states were assigned: Wake, REM sleep and NREM sleep. Wake consists of visible EEG theta activity and high EMG activity, REM sleep consists of clear, sustained EEG theta activity and phasic muscle twitches on a background of low EMG, NREM sleep consists of high amplitude, synchronized EEG and low EMG activity.

EEG and EMG signals were recorded for 24 h of baseline after which the animals were unhooked from the recording system and single prolonged stress (SPS, described below) was performed. Following SPS, animals were reconnected to the recording system and seven subsequent days were recorded and scored. Data collected after SPS were compared to the baseline recording day using one-way repeated measures ANOVA followed by Bonferroni post hoc comparisons of each day to the baseline. Sleep states were quantified as an average duration spent in state per hour (in seconds) over the Light Phase (ZT0-12) and Dark Phase (ZT12-24). Sleep architecture quantified the mean bout length and mean bout number per hour for each of the states discussed above and was averaged over the sleep/active phase and analyzed using one-way repeated measures ANOVA followed by Bonferroni post hoc comparisons of each day to the baseline.

### Single prolonged stress

Single prolonged stress was performed as previously published^16,17^. Briefly, animals were exposed to 3 successive stressors at the start of the Dark Phase (lights off-ZT12). First, physical restraint was performed for 2 h in custom built plexi-glass restraining devices. Next, the animals were placed in a (25 × 17 × 16 i in.) plastic bin containing 21–24 °C water and were forced to swim in groups of 6–8 for 20 min. Following a 15-min recuperation period in a towel-lined bin, the animals were exposed to 60 ml of ether vapors in a 2000 cc isolation chamber until fully anesthetized (< 5 min). After which the animals were returned to their EEG/EMG recording-cage where they were isolated for the following seven days.

### Fear conditioning, fear extinction, and extinction recall

At the conclusion of the EEG/EMG recording, fear conditioning experiments were conducted. Fear conditioning, extinction, and extinction recall were performed as previously published^17,20^. All fear conditioning/extinction/extinction recall experiments were performed in four identical Noldus fear conditioning chambers (Wageningen, the Netherlands) (12″W × 10″D × 12″H) containing a Shock Floor with current carrying metal bars, a wall-mounted speaker and in-chamber UV and white lighting. Test cages were housed in sound-attenuating boxes. Tones (2000 Hz, 80 dB) were delivered via speakers mounted in the housing of the test cages and controlled by data acquisition software (Noldus Ethovision). Ceiling mounted cameras recorded behavior for analysis and Noldus Ethovision software was used to quantify freezing levels.

Two unique contexts were created by manipulating olfactory and visual cues. Context A consisted of 50 ml of 1% acetic acid solution placed in a small dish next to the test cage using standard lighting conditions of the above mentioned housing boxes. Context B consisted of 50 ml a 1% ammonium hydroxide solution placed in a small dish above the test cage along with a checkerboard patterned paper placed on the chamber walls to alter the visual context.

Fear conditioned animals were exposed to five, 1 mA, 1-s foot-shocks paired with the cessation of a 10 s 80 dB tone in Context B. The first tone was presented 180 s after the animal was placed in the test cage and the subsequent tones occurred with a 60 s inter-tone interval. For all phases, baseline freezing was assessed for 180 s prior to the presentation of any tones, and the inter-tone interval was 60 s. 60 s after the last tone, animals were removed to their home cages. Fear extinction was conducted 24 h after fear conditioning and was performed in a distinctly different context (Context A). Fear extinction consisted of presentation of 30 tones (60 s inter-tone interval), without the paired foot-shock. Extinction recall was assessed 24 h after extinction and consisted of the animals being placed back into the fear extinction context (Context A) for 10 tones (60 s inter-tone interval), again without foot shock.

The percent time spent immobile (freezing) within each 60 s long block was calculated by Ethovision software. Statistical comparison of time spent freezing was made using two-tailed Students t-test via Graphpad Prism software statistical package. Average freezing levels between groups were compared on fear conditioning, extinction, and extinction recall days.

### Experimental timeline

#### Experiment 1

**Figure 1:**
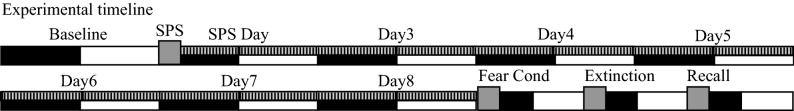
Experimental timeline. 24 h of baseline EEG/EMG activity was recorded on Baseline Day (12/12, Dark/Light schedule) in animals injected with rAAV-MCH-ChR2-EGFP. At the conclusion of the baseline recording (ZT12, the onset of the Dark Phase), SPS was delivered to all animals (gray box). Rats were immediately returned to the recording chambers and were either non-stimulated (represented by the continued LD schedule below), or optogenetically stimulated (vertical bars). Both groups of animals were recorded for the subsequent 7 days (SPS Day-Day8). At the conclusion of the recordings, all animals were unhooked and tested for fear-associated memory impairments using fear conditioning, extinction and recall (gray boxes).

Rats (n = 7) were injected with rAAV-MCH-ChR2-EGFP and tethered to stimulation/recording equipment baseline EEG/EMG signals were measured for 24 h from ZT12-ZT12 and followed by SPS (ZT12). After SPS, the animals were reconnected to the recording system and the optogenetic stimulation was not activated. Comparisons of duration in vigilance state were made vs. baseline (pre-trauma) recordings. Fear conditioning experiments proceeded at the conclusion of the EEG/EMG recording period (Fig. 1). Additional control experiments were performed at this time and compared baseline sleep in non-ChR2 expressing animals (rAAV-MCH-EYFP, optogenetically stimulated and non-stimulated) to non-AAV injected and ChR2 expressing animals (rAAV-MCH-ChR2-EYFP, optogenetically stimulated and non-stimulated) were made at this stage as per Blanco Centurion^25^.

#### Experiment 2

As in Experiment 1, rats (n = 9) were injected with rAAV-MCH-ChR2-EGFP and tethered as described above. Baseline EEG/EMG signals were measured for 24 h from ZT12-ZT12, followed immediately by SPS (ZT12). After SPS, the animals were reconnected to the recording system and the optogenetic stimulation was activated. Comparisons of duration in vigilance state were made vs. baseline (pre-trauma) recordings. Fear conditioning experiments proceeded at the conclusion of the EEG/EMG recording period.

## Results

### Experiment 1

As a proof of concept of optogenetic stimulation increasing sleep time, same as in Blanco Centurion^25^, we first made a comparison of baseline sleep duration from non-optogenetically-stimulated and stimulated rAAV-MCH-ChR2-EGFP injected animals to animals that received a control rAAV vector, and to non-virus injected animals. We found no difference in baseline sleep in either the control rAAV expressing or non-stimulated ChR2 expressing animals from non-virus injected animals, indicating that injection of virus alone had no effect on baseline sleep duration. Additionally, the control rAAV expressing animals showed no changes in sleep from their baseline when optically stimulated. Optogenetic stimulation of the rAAV-MCH-ChR2-EGFP expressing animals resulted in increased REM sleep during both the Dark and Light phase and increased Dark phase NREM sleep increases that replicate the results from previously published work with these two viruses^25^. As a result of these confirmatory control studies, we focused our subsequent efforts on determining the contribution of SPS with and without optogenetic stimulation in the ChR2 expressing animals.

SPS alters sleep and impairs fear-associated memory when given at “lights on” (ZT0)^17^. However, the impact of SPS on sleep and fear-associated memory processing when the trauma is presented at “lights off” (ZT12) was previously unexamined. Therefore, in order to determine the time-course of trauma-induced sleep alterations and fear-associated memory impairments, we exposed animals to SPS at the transition from “lights on-to lights off” (ZT 12). Our previous work showed that REM sleep was significantly increased in the Dark Phase during the 12 h after SPS trauma exposure and that NREM sleep duration was reduced during the Dark Phase 4–7 days after trauma^17^. Although time-of-day effects have been shown for other learning and memory processes, no work has been performed to determine the time-of-day effects of trauma exposure that leads to PTSD.

**Figure 2:**
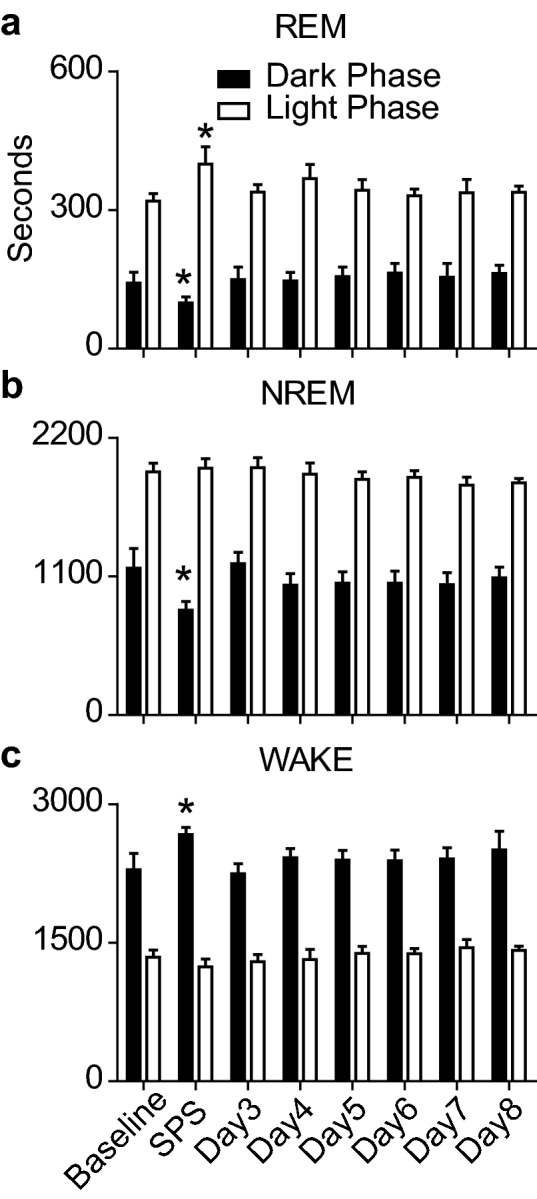
Sleep/Wake responses before and after SPS. Each bar represents the duration (seconds) spent in (**a**) REM, (**b**) NREM, and (**c**) Wake, averaged per hour over the 12 h Dark (or active) Phase (black bars), and the 12 h Light (or sleep) Phase (white bars) over the course of the 8 day experiment. One-way ANOVA was conducted to assess the effects of SPS on (**a**) REM, (**b**) NREM, and (**c**) WAKE duration over the Dark and Light phases. (**a**) REM sleep was significantly altered by SPS exposure during both the Dark Phase (F(7,55) = 3.3, p = 0.007) and Light Phase (F(7,55) = 1.6, p = 0.05). Bonferroni *post hoc* comparisons revealed that REM sleep duration was significantly reduced during the Dark Phase on the SPS day (M = 99.1 ± 13 s) compared to baseline (M = 142.0 ± 23.6 s). REM sleep during the Light Phase on the SPS day (M = 399.9 ± 37.2 s) was significantly increased compared to baseline (M = 319.5 ± 16.4 s). (**b**) NREM sleep was significantly altered by SPS exposure during the Dark Phase (F(7,55) = 5.79, p < 0.0001), but not during the Light Phase (F(7,55) = 1.52, p = 0.19). Bonferroni post hoc comparisons revealed that NREM sleep duration was reduced during the Dark Phase on the SPS day (M = 833.5 ± 67.5 s) compared to baseline (M = 1168.0 ± 154.4 s). (**c**) WAKE was significantly altered by SPS exposure during the Dark Phase (F(7,55) = 2.74, p = 0.019), but not during the Light Phase (F(7,55) = 1.79, p = 0.12). Bonferroni post hoc comparisons revealed that WAKE duration was significantly increased during the Dark Phase on the SPS day (M = 2670 ± 77.5 s) compared to baseline (M = 2290.0 ± 177.2 s) (*p < 0.05).

When delivered at ZT12, SPS resulted in a 43 min reduction in REM sleep during the Dark Phase following trauma, and 12 h later, during the subsequent Light Phase, SPS resulted in a 80 min increase in REM sleep (Fig. 2a) compared to their respective baseline measures. NREM sleep was reduced by 334.5 min during the Dark Phase immediately following SPS exposure and was unchanged from baseline for the remainder of the experiment (Fig. 2b). SPS exposure increased Wake duration by 380 min during the Dark Phase immediately following the trauma (Fig. 2c).

**Figure 3:**
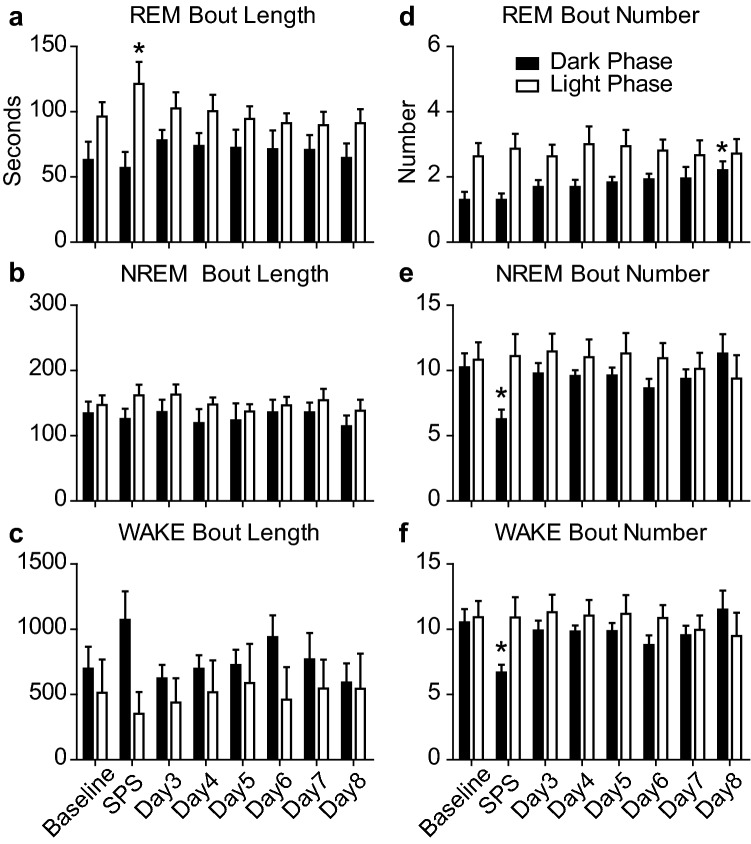
Sleep architecture before and after SPS. Each bar represents the average bout length (**a**–**c**) or average bout number (**d**–**f**) per hour over the 12 h Dark (or active) Phase (black bars), and the 12 h Light (or sleep) Phase (white bars) over the course of the 8 day experiment. One-way ANOVA was conducted to compare the effects of SPS on REM, NREM, and WAKE bout length and number over the Dark and Light phases. (**a**) REM bout length was significantly altered by SPS exposure during the Light Phase (F(7,55) = 3.38, p = 0.006) but not the Dark Phase (F(7,55) = 0.65, p = 0.71). Bonferroni post hoc comparisons revealed that REM sleep bout length was significantly increased during the Light Phase on the SPS day (M = 121.3 ± 16.8 s) compared to baseline (M = 96.3 ± 10.9 s). (**b**) NREM bout length was not significantly altered by SPS exposure during the Light Phase (F(7,55) = 0.88, p = 0.53) or Dark Phase (F(7,55) = 0.90, p = 0.52). (**c**) WAKE bout lengths were similarly not altered by SPS exposure during the Light Phase (F(7,55) = 1.79, p = 0.11) or Dark Phase (F(7,55) = 1.75, p = 0.12). (**d**) REM bout number was significantly altered by SPS exposure during the Dark Phase (F(7,55) = 2.81, p = 0.017) but not the Light Phase (F(7,55) = 0.54, p = 0.80). Bonferroni post hoc comparisons revealed REM sleep bout number was significantly increased during the Dark Phase on day 8 (M = 2.2 ± 0.22) compared to baseline (M = 1.3 ± 0.24). (**e**) NREM bout number was significantly altered by SPS exposure during the Dark Phase (F(7,55) = 3.59, p = 0.004) but not the Light Phase (F(7,55) = 0.47, p = 0.85). Bonferroni post hoc comparisons revealed NREM sleep bout number was significantly reduced during the Dark Phase immediately following SPS (M = 6.3 ± 0.72) compared to baseline (M = 10.24 ± 1.1). (**f**) WAKE bout number was significantly altered by SPS exposure during the Dark Phase (F(7,55) = 3.39, p = 0.006) but not the Light Phase (F(7,55) = 0.54, p = 0.80). Bonferroni post hoc comparisons revealed WAKE bout number was significantly decreased during the Dark Phase on the SPS day (M = 6.68 ± 0.6) compared to baseline (M = 10.54 ± 1.0) (*p < 0.05).

Sleep architecture was significantly altered by SPS exposure at ZT12. REM bout length increased by 25 min during the Light Phase on the SPS exposure day as compared with baseline (Fig. 3a). NREM and Wake bout length was unchanged compared to baseline at any point during the experiment (Fig. 3b,c). REM bout number increased day over day during the Dark Phase of the experiment, reaching statistical significance on Day 8 (Fig. 3d). SPS exposure resulted in a 38% and 36% reduction in the number of NREM and Wake bouts, respectively, during the Dark Phase compared to baseline (Fig. 3e,f).

### Experiment 2

**Figure 4:**
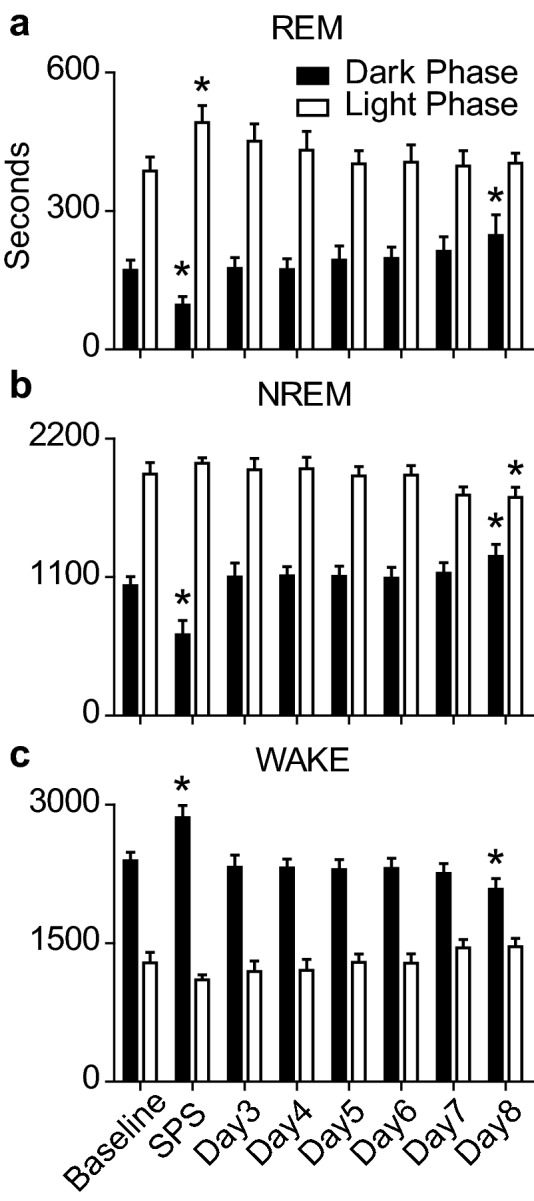
Sleep profile of SPS exposed animals with concurrent optogenetic stimulation of MCH containing cells of the lateral hypothalamus. Sleep/Wake responses before and after SPS with concurrent, 24 h, 10 Hz, 1 min on, 4 min off repeating optogenetic stimulation. Each bar represents the duration (seconds) spent in (**a**) REM, (**b**) NREM, and (**c**) Wake, averaged per hour over the 12 h Dark Phase (black bars), and the 12 h Light Phase (white bars) over the course of the 8 day experiment. One-way ANOVA was conducted to compare the effects of SPS and optogenetic stimulation on (**a**) REM, (**b**) NREM, and (**c**) WAKE duration over the Dark and Light phases. (**a**) REM sleep was significantly altered by SPS exposure and optogenetic stimulation during both the Light Phase (F(7,71) = 2.31, p = 0.04) and Dark Phase (F(7,71) = 6.14, p < 0.0001). Bonferroni post hoc comparisons revealed that REM sleep duration was significantly increased during the Light Phase on the SPS exposure day (M = 491.7 ± 37.2 s) compared to baseline (M = 386.7 ± 30.5 s). REM sleep during the Dark Phase was significantly decreased on the SPS exposure day (M = 96.1 ± 18.9 s) compared to baseline (M = 171.9 ± 21.9 s) and showed additional, post-SPS, daily increases in REM sleep duration that reached statistical difference from baseline on Day 8 (M = 247.2 ± 44.6 s). (**b**) NREM sleep was significantly altered by SPS exposure and optogenetic stimulation during the Dark Phase (F(7,71) = 12.14, p < 0.0001), and Light Phase (F(7,71) = 4.76, p = 0.0003). Bonferroni post hoc comparisons revealed that NREM sleep duration was significantly decreased during the Dark Phase immediately following SPS (M = 641.9 ± 114.6 s) compared to baseline (M = 1034.0 ± 70.6 s) and showed increased NREM sleep time on Day 8 (M = 1267.0 ± 95.1 s). NREM sleep duration was significantly decreased during the Light Phase on Day 8 (M = 1733 ± 62.3 s) compared to baseline (M = 1918.0 ± 91.4 s). (**c**) WAKE was significantly altered by SPS exposure and optogenetic stimulation during the Dark Phase (F(7,71) = 14.27, p < 0.0001), but not during the Light Phase (F(7,71) = 18.12, p = 0.0002). Bonferroni post hoc comparisons revealed that WAKE duration was significantly increased during the Dark Phase on the SPS exposure day (M = 2862.0 ± 131.2 s) compared to baseline (M = 2394.0 ± 91.2 s) and reduced by Day 8 (M = 2086.0 ± 114.0 s). There were no significant changes from baseline during the Light Phase (*p  < 0.05).

The overarching goal of this work was to examine if increasing sleep via optogenetic stimulation of MCH containing cells would restore fear-associated memory functioning in SPS trauma-exposed rats. SPS with optogenetic stimulation (Opto Stim) resulted in a 75 min reduction in REM sleep during the Dark Phase following trauma. 12 h later, during the Light Phase, REM sleep increased 105 min compared to baseline. In addition, REM sleep showed daily increases during the Dark Phase of the experiment that reached statistical significance by Day 8 (Fig. 4a). NREM sleep was reduced by 392 min during the Dark Phase immediately following SPS exposure and, similar to REM, showed daily increases over the Dark Phase that reached statistical significance on Day 8 (Fig. 4b). SPS exposure resulted in an increase of 468 min of Wake compared to baseline during the Dark Phase immediately following the trauma and a reduced duration spent awake on Day 8 that concurs with the increases in REM and NREM sleep duration on that day (Fig. 4c).

**Figure 5:**
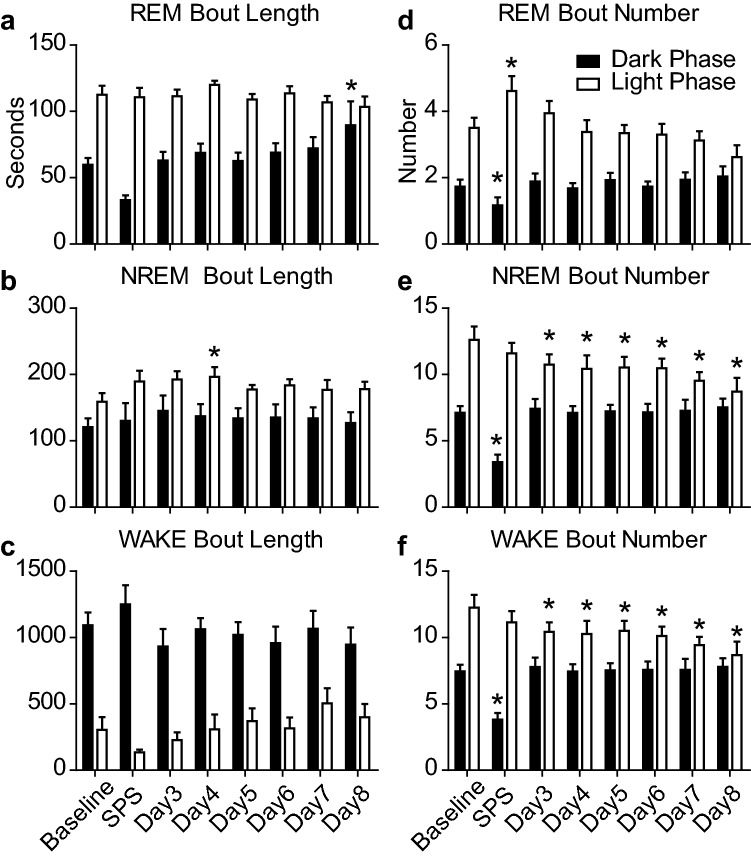
Sleep architecture alterations before and after SPS with combined optogenetic stimulation of MCH containing cells of the lateral hypothalamus. Each bar represents the average bout length (**a**–**c**) or average bout number (**d**–**f**) per hour over the 12 h Dark (or active) Phase (black bars), and the 12 h Light (or sleep) Phase (white bars) over the course of the 8 day experiment. One-way ANOVA was conducted to compare the effects of SPS and optogenetic stimulation on REM, NREM, and WAKE bout length and number over the Dark and Light phases. (**a**) REM bout length was significantly altered by SPS exposure during the Dark Phase (F(7,71) = 4.65, p = 0.0004) but not the Light Phase (F(7,71) = 0.81, p = 0.58). Bonferroni post hoc comparisons revealed that REM sleep bout length was significantly increased during the Dark Phase on the Day 8 (M = 89.57 ± 18.1 s) compared to baseline (M = 59.8 ± 5.0 s). (**b**) NREM bout length was significantly altered by SPS exposure during the Light Phase (F(7,71) = 1.8, p = 0.01) but not during the Dark Phase (F(7,71) = 0.61, p = 0.74). Bonferroni post hoc comparisons of light phase NREM bout lengths were significantly increased on Day 4 (M = 196.3 ± 14.9) compared to baseline (M = 158.9 ± 12.9). (**c**) WAKE bout lengths were altered by SPS exposure during the Light Phase (F(7,71) = 4.9, p = 0.0002) but not during the Dark Phase (F(7,71) = 1.4, p = 0.21). Bonferroni post hoc comparisons of Light Phase WAKE bout length revealed no significant changes from baseline. (**d**) REM bout number was significantly altered by SPS exposure during the Dark Phase (F(7,71) = 3.9, p = 0.002) and the Light Phase (F(7,71) = 5.3, p = 0.0001). Bonferroni post hoc comparisons revealed REM sleep bout number was significantly increased during the Light Phase on the SPS exposure day (M = 4.6 ± 0.45) compared to baseline (M = 3.5 ± 0.31) and REM sleep bout number was significantly reduced during the Dark Phase on the SPS exposure day (M = 1.2 ± 0.24) compared to baseline (M = 1.7 ± 0.21). (**e**) NREM bout number was significantly altered by SPS exposure during the Dark Phase (F(7,71) = 5.9, p < 0.0001) and the Light Phase (F(7,71) = 9.9, p < 0.0001). Bonferroni post hoc comparisons revealed NREM sleep bout number was significantly reduced during the Dark Phase immediately following SPS (M = 3.4 ± 0.56) compared to baseline (M = 7.1 ± .49) and during the Light Phase, NREM bout number was significantly reduced from baseline on days 3–8. (**f**) WAKE bout number was significantly altered by SPS exposure during the Dark Phase (F(7,71) = 10.5, p = 0.0001) and the Light Phase (F(7,71) = 5.2, p = 0.0001). Bonferroni post hoc comparisons revealed WAKE bout number was significantly decreased during the Dark Phase on the SPS day (M = 3.8 ± 0.5) compared to baseline (M = 7.5 ± 0.5) and significantly decreased from baseline on days 3–8) (*  p < 0.05).

Sleep architecture was significantly altered by SPS exposure and concurrent optogenetic stimulation. REM bout length was significantly increased during the Dark Phase on the Day 8 (Fig. 5a). NREM bout length was significantly increased during the Light Phase on Day 4 above baseline (Fig. 5b). Wake bout length was unchanged compared to baseline at any point during the experiment (Fig. 5c). REM bout number decreased during the Dark Phase and increased during the subsequent Light Phase on the SPS exposure day (Fig. 5d). SPS exposure significantly reduced NREM and Wake bout number during the Dark Phase and resulted in a subsequent decrease in bout number on days 3–8 (Fig. 5e,f).

**Figure 6:**
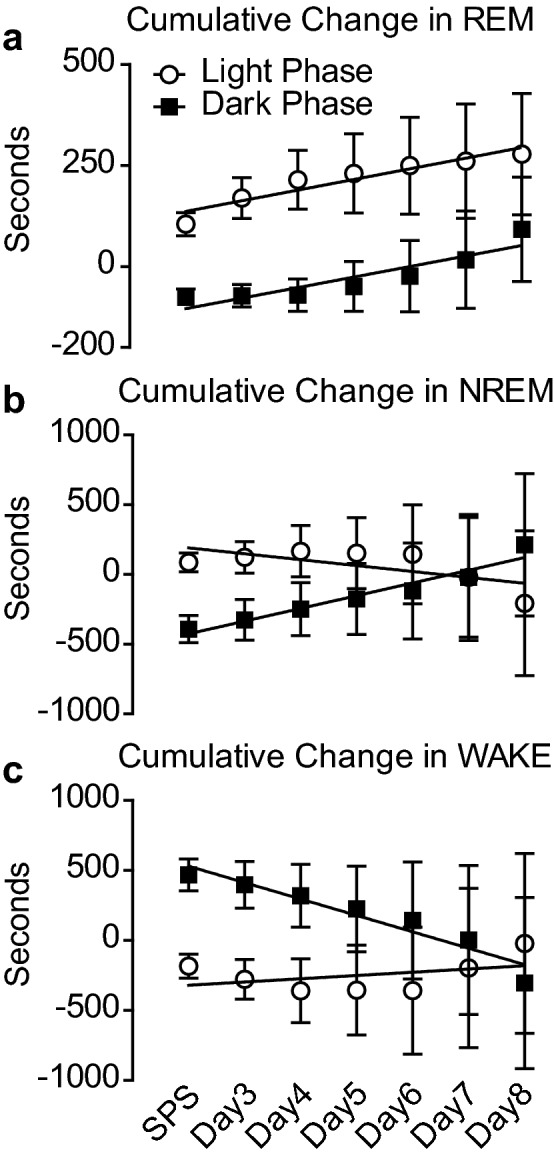
Continuous optogenetic stimulation of MCH containing cells of the LH following SPS results in cumulative changes in sleep/wake. The cumulative time spent in sleep/wake stage compared to baseline is shown for each day over the 12 h Dark Phase (black squares), and the 12 h Light (or sleep) Phase (white circles) over the course of the experiment. (**a**) Linear regression analysis revealed that REM sleep increased during both the Dark Phase (r^2^ = 0.83, p = 0.0045) and the Light Phase (r^2^ = 0.89, p = 0.0013) over the course of the experiment. (**b**) NREM sleep showed cumulative sleep time increases specific to the Dark Phase (r^2^ = 0.94, p = 0.0004) whereas, NREM sleep time was unchanged during the Light Phase (r^2^ = 0.46, p = 0.09). (**c**) Similar to the cumulative change in NREM, the cumulative changes in WAKE showed a significant reduction during the Dark Phase over the course of the experiment (r^2^ = 0.92, p = 0.007) whereas WAKE was unchanged during the Light Phase of the experiment (r^2^ = 0.15, p = 0.37).

To visualize if optogenetic stimulation was effective at increasing sleep over the course of the experiment, we plotted the cumulative change in sleep for the Dark and Light Phases independently over the course of the experiment (as a change from baseline). Optogenetic stimulation was sufficient to cause daily increases in REM sleep during both the Dark and Light Phases as the slopes for both lines are significantly non-zero (Fig. 6a). NREM sleep was significantly increased over the course of the experiment only during the Dark Phase. The slope of the cumulative change in NREM sleep during the Light Phase was not different from zero (Fig. 6b). Similar to the NREM sleep duration, the effect of optogenetic stimulation of MCH cells resulted in a significant decrease in Wake over the Dark Phase of the experiment, but not during the Light Phase (Fig. 6c). Similarly, when optogenetically stimulated animals were cumulatively compared to control (non-optogenetically stimulated) SPS treated animals, a similar pattern of increased sleep emerged (Figure S2).

**Figure 7:**
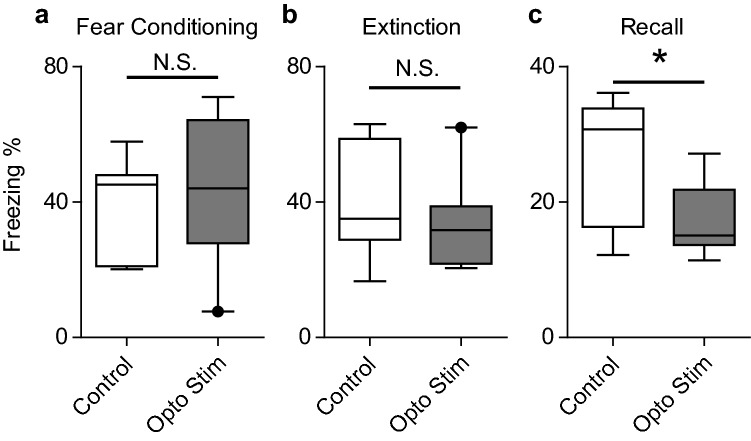
Fear-associated memory impairments are attenuated by optogenetic stimulation of MCH cells. Animals injected with rAAV-MCH-ChR2-EGFP were exposed to SPS and EEG/EMG recordings were made for 7 subsequent days. Control animals (those in Figs. 2 and 3) did not receive optogenetic stimulation, while another group (those in Figs. 4, 5, 6) received 7 days of optogenetic stimulation. At the conclusion of the stimulation, all animals underwent fear testing as described in the methods section. (**a**) After the fear conditioning day, optogenetically stimulated (Opto Stim) animals (M = 43.8, SEM = 6.9) and non-stimulated (Control) animals (M = 39.8, SEM = 5.3) showed no difference in freezing (t(14) = 0.44, p = 0.67). (**b**) 24 h later, fear memory extinction was examined and, again, Opto Stim (M = 32.6, SEM = 4.4) and control animals (M = 41.5, SEM = 6.8) had similar freezing levels (t(14) = 1.14, p = 0.27). (**c**) 48 h after initial fear conditioning, fear recall was examined and Opto Stim animals (M = 17.4, SEM = 1.8) showed significantly reduced freezing compared to non-stimulated Control animals (M = 26.0, SEM = 3.7) (t(14) = 2.3, p = 0.04) (*p <  0.05).

Fear-associated memory impairments are associated with SPS exposure and fear extinction recall impairments have been correlated to PTSD-like severity in this animal model. Therefore, given that optogenetic stimulation is sufficient to increase REM sleep during both the Light and Dark phase and NREM sleep during the Dark Phase following SPS we examined if this increasing sleep could rescue the cognitive deficits typically seen following SPS trauma exposure. At the conclusion of the EEG/EMG recordings, animals were unhooked from the recording tethers and exposed to the fear conditioning/extinction/recall protocol described in the methods to determine the effect of sleep restoration on the development of fear-associated memory impairments. Data were compared between control and optogenetically stimulated experimental animals using Students t-test. No differences were detected following the initial 5 tone/shock pairings in context B of fear conditioning (Fig. 7a) nor during the 30 trial, tone only presentations in context A of the extinction period (Fig. 7b). During the 10 trial, tone only presentations in context A of the Recall phase of the test (Fig. 7c), optogenetically stimulated animals showed a 33% reduction in freezing behavior compared to Control animals.

## Discussion

The timing of trauma exposure is unpredictable and trauma-exposed human populations show variability in their resulting trauma-exposure-dependent sleep phenotypes^27–29^. Therefore, it is likely that the time-of-day plays a role in the manifestation of trauma-induced changes in sleep. Trauma-type or trauma-intensity may also be confounding factors in human studies. However, our use of SPS alleviates variability that may be introduced due to these factors; further solidifying a role for time-of-day in mediating trauma-exposure-dependent sleep phenotypes. The data presented from control animals (Figs. 2, 3) show an acute increase in REM sleep, 12 h after SPS exposure. This 12 h delay in increased REM sleep is a consistent phenotype across this study and our previous work^17^. However, sleep phenotypic differences remain between these data and our previous report. For instance, our previous work identified a significant SPS-induced, Dark Phase reduction of NREM sleep starting 4 days after SPS. These data also identified increased Wake activity during the Dark Phase concurrent with the decrease in NREM sleep. These results are lacking in the current experiments. It is possible that SPS results in acute and chronic sleep changes when the trauma is presented at ZT0 and only acute changes in sleep when presented at ZT12. A close examination of the data in Fig. 2 shows that NREM and Wake activity is trending towards those values seen when SPS is presented at ZT0, but the data are not statistically significant. It is possible that the development of this chronic sleep phenotype requires more time to develop when trauma occurs at ZT12. Sleep in the SPS model has not been examined past 7 days post-SPS and future studies should examine the long-term impact of SPS trauma exposure on sleep.

Consistent with a prior report, baseline sleep is not altered by expression of GFP control virus or ChR2 in the absence of stimulation^25^. Therefore, we chose to examine sleep and fear-associated memory in ChR2 expressing animals that were either stimulated or not stimulated. Non-virus expressing animals and GFP expressing control animals were therefore not examined over the course of the experiment. However, in our laboratory, optogenetic stimulation of MCH cells was sufficient to increase REM sleep following SPS during both the Light and Dark Phases of the circadian day (Figs. 4, 5, 6). Interestingly, NREM sleep showed significant cumulative increases from baseline only during the Dark Phase, but not during the Light Phase. Although curious, these data replicate findings by Blanco Centurion et al.^25^ showing that 10 Hz optogenetic stimulation was effective to increase REM sleep during the Light and Dark Phase and NREM sleep only during the Dark Phase. This study examined optogenetic stimulation over 7 days and showed that although the increases in REM and NREM sleep from optogenetics may have been incremental, but were still sufficient to improve function on a fear-associated memory task. It is possible that long-term optogenetic stimulation may reach a ceiling where further stimulation does not enhance sleep any longer, but our data indicate that for at least 7 days it is effective. Further, although we did not verify expression of the viral construct in every animal tested, the behavioral changes we see in sleep and in response to fear recall are robust enough to suggest that expression of the channel was widespread and effective at improving fear-associated learning impairments.

For these studies, our primary outcome measure was to examine fear-associated memory impairment (as measured by freezing in response to foot shock and/or a conditioned stimulus tone) and compare optogenetically stimulated animals to their non-stimulated controls. Freezing during the fear-conditioning trial was not different between the optogenetically stimulated and non-stimulated groups. Freezing on the extinction day, was again, not different between the optogenetically stimulated and non-stimulated groups. However, on the fear-recall day, optogenetically stimulated animals showed reduced freezing compared to their non-stimulated controls, indicating that they consolidated the memory of the extinction better and did not display the fear-extinction deficits that typify SPS exposure^17,20,30^. We interpret these results to mean that post-SPS sleep improvements were sufficient to improve subsequent fear-associated memory processing. These results are in line with findings in trauma exposed humans that show that poor sleep increases the likelihood of acquiring PTSD^31^ and that improved sleep aids in the processing of emotional stimuli^32^ and for the elimination of intrusive memories^33,34^. These results are interesting due to the well described contribution of sleep in memory processing^35–38^ and in the molecular mechanisms that regulate memory^39^. Post-training sleep deprivation has been shown to impair memory consolidation for associative tasks^40^, contextual fear memory processing^41^, and fear avoidance^42,43^. In our model, we have improved sleep after SPS and have presumably consolidated the memory of the original trauma and yet have improved function on a subsequent fear-associated memory task. It is possible, therefore, that sleep following trauma exposure is performing a different function than sleep after a fear-associated task that does not result in PTSD-like phenotypes. Therefore, a distinction must be made between sleep alterations that accompany trauma exposure (SPS in this case) that results in long term impairment of neurobiological systems, and fear-associated memory tasks (fear conditioning) that are designed to probe how an animal deals with a stressor that has no long lasting impact on the underlying biology. In our studies, the optogenetic stimulation was discontinued prior to the fear-associated memory tasks and therefore the impact of post-fear-conditioning optogenetic stimulation on fear-associated memory processing remains unknown. However, our discontinuation of optogenetic stimulation at this time may have serendipitously revealed a window of time that is sufficient to improve subsequent fear-memory processing after trauma exposure since these animals did show reduced freezing on the recall day. Interestingly, there is also a trend towards better performance on the extinction task in optogenetically stimulated animals. Although not significant, these data suggest that extinction memory related molecular mechanisms tied to sleep need to be further explored. Additional future studies should include an examination of post fear-conditioning sleep and the subsequent result of post fear-conditioning sleep deprivation and/or optogenetic sleep improvements on fear-recall outcomes in trauma-exposed and control animals.

The optogenetically stimulated animals showed more REM sleep and better performance on a subsequent fear-associated memory task. These data are consistent with the *sleep to forget and sleep to remember* model of emotion processing posed by Walker et al.^44,45^. This model posits that REM sleep provides an optimal neurobiological setting for the reduction of affective tone for any given emotional memory while preserving the memory itself. This theory states that REM sleep is beneficial to cognitive processing of emotional memories and that REM sleep functions to reduce the affective tone of an emotionally enriched memory. Therefore, the optogenetic stimulation may have reset trauma exposed animals back to a normal level of functioning. Conversely, our previous data^17^ showed that impairment on a fear-associated memory task was correlated to increased REM sleep immediately following trauma exposure. Additional human studies have also linked sleep to the preservation of emotional memories^46^. These are seemingly contradictory data, however, it is possible that timing and duration of sleep helps to determine specific outcomes. For example, increased REM immediately after trauma may increase memory consolidation of the traumatic event, while continued REM sleep increases help to remove the affective tone from that same experience (via *sleep to forget and sleep to remember* mechanisms). REM specific and total sleep deprivations following trauma exposure are required to fully assign function to this sleep state in improving subsequent fear-associated memory processing. Lastly, our current data show that NREM sleep increased over the Dark Phase of the experiment. It is possible that NREM sleep is the critical component in fear-associated memory performance following traumatic stress. These data suggest that further work is required to isolate the components of REM or NREM sleep responsible for this behavioral restoration.

For years, researchers have argued that therapeutics designed to improve sleep may be required for the restoration of function in PTSD^2^. Yet, a primary treatment for PTSD remains the SSRI class of drugs which have been shown to reduce or abolish REM sleep altogether^47,48^. Many other drug classes are reported to decrease REM sleep (e.g., antiarrhythmics, alcohol, benzodiazepines, corticosteroids, diuretics and of course, caffeine), while only a handful are accompanied by REM sleep increases in humans. For example, in normal subjects, melatonin increased percent of time in REM and REM episode duration compared to their baseline^49^. This effect of melatonin administration was also confirmed in subjects with REM sleep disruptions^50^. Dopamine, norepinephrine and serotonin reuptake inhibitors (reserpine and bupropion), alpha adrenergic blockers (thymoxamine and mesoridazine) selective serotonin antagonists (nefazedone and ritanserine), and cholinergic agonists (carbachol and bethanechol) all increase REM sleep^51–56^. These therapeutics may provide promising ways to mitigate the adverse symptoms of PTSD.

Non-pharmacological approaches to decreasing REM are uncommon, while increasing ambient temperature and humidity increases REM sleep in rodents^57^, this does not translate to humans and can disrupt NREM and REM sleep^58,59^.

Increasing sleep was sufficient to rescue trauma-related fear-associated memory defects, however, the downstream physiological mechanisms for this result are unknown. Additionally, people with PTSD show impairment in memory recall for non-fear-based tasks that may also require sleep to function properly. Future studies are required to examine the contribution of sleep improvements on non-fear based cognitive function and to determine how optogenetic manipulation alters the underlying neuro-circuitry involved in fear, memory, and cognition.

## Supplementary information

Supplementary Figure Legend.

Supplementary Figure S1.

Supplementary Figure S2.

## Data Availability

Data will be made available upon request.

## Abbreviations

PTSD: Post-traumatic stress disorder
SPS: Single prolonged stress
EEG: Electroencephalogram
EMG: Electromyogram
REM: Rapid eye movement sleep
NREM: Non rapid eye movement sleep
SSRI: Selective serotonin reuptake inhibitor
