# Supplementary material for: Optogenetic sleep enhancement improves fear-associated memory processing following trauma exposure in rats
Source: Sci Rep. 2020 Oct 22;10:18025. doi: 10.1038/s41598-020-75237-9 (PMC7581760; doi:10.1038/s41598-020-75237-9)
Supplement: Supplementary file 1 — Supplementary Figure Legend. [file 41598_2020_75237_MOESM1_ESM.pdf]

Supplemental Data

Optogenetic sleep enhancement improves fear-associated memory processing following trauma exposure in rats

Sleep improvements improve fear-associated memory

Christopher J. Davis Ph.D.[1], *William M. Vanderheyden Ph.D.[1]

[1] WSU Health Sciences Spokane

Elson S. Floyd College of Medicine

Department of Biomedical Sciences

Pharmaceutical and Biomedical Sciences

Room 213/Lab 230

412 E. Spokane Falls Blvd

Spokane, WA 99202 USA

*Corresponding Author:

William M. Vanderheyden Ph.D.

w.vanderheyden@wsu.edu

(Lab) 509-368-0690

Figure S1. Viral transfection of the rAAV-MCH-ChR2-EGFP was confirmed by fluorescent microscopy of paraformaldehyde perfused, frozen, and cryostat sectioned brain slices of the lateral hypothalamus. The images shows EGFP expression throughout the LH that mirrors the expression of this virus as previously published.

Figure S2. Comparison of cumulative sleep changes in optogenetically stimulated animals to non-stimulated controls. The cumulative time spent in sleep/wake stage is shown for each day over the 12 hour Dark Phase (black squares), and the 12 hour Light (or sleep) Phase (white circles) over the course of the experiment. A) Linear regression analysis revealed that REM sleep increased during both the Dark Phase ($r^2 = 0.95$, $p = 0.0002$) and the Light Phase ($r^2 = 0.99$, $p < 0.0001$) in the optogenetically stimulated animals over the course of the experiment. B) NREM sleep showed cumulative sleep time increases specific to the Dark Phase ($r^2 = 0.78$, $p = 0.0087$) whereas, NREM sleep time was unchanged during the Light Phase ($r^2 = 0.03$, $p =0.69$). C) The cumulative changes in WAKE showed a significant reduction during the Dark Phase over the course of the experiment ($r^2 = 0.80$, $p = 0.0068$) and the Light Phase of the experiment ($r^2 = 0.84$, $p = 0.004$).
